# How to effectively obtain informed consent in trauma patients: a systematic review

**DOI:** 10.1186/s12910-019-0347-0

**Published:** 2019-01-23

**Authors:** Yen-Ko Lin, Kuan-Ting Liu, Chao-Wen Chen, Wei-Che Lee, Chia-Ju Lin, Leiyu Shi, Yin-Chun Tien

**Affiliations:** 1 Division of Trauma and Surgical Critical Care, Department of Surgery, Kaohsiung Medical University Hospital, Kaohsiung Medical University, Kaohsiung, Taiwan; 2 Department of Medical Humanities and Education, College of Medicine, Kaohsiung Medical University, Kaohsiung, Taiwan; 3 Department of Emergency Medicine, College of Medicine, Kaohsiung Medical University, Kaohsiung, Taiwan; 4 College of Nursing, Kaohsiung Medical University, Kaohsiung, Taiwan; 5 Department of Health Policy and Management, Bloomberg School of Public Health, Johns Hopkins University, Baltimore, MD 21205 USA; 6 Department of Orthopedics, Kaohsiung Medical University Hospital, Kaohsiung Medical University, Kaohsiung, Taiwan; 7 Department of Orthopedics, College of Medicine, Kaohsiung Medical University, Kaohsiung, Taiwan

**Keywords:** Informed consent, Trauma, Ethics

## Abstract

**Background:**

Obtaining adequate informed consent from trauma patients is challenging and time-consuming. Healthcare providers must communicate complicated medical information to enable patients to make informed decisions. This study aimed to explore the challenges of obtaining valid consent and methods of improving the quality of the informed consent process for surgical procedures in trauma patients.

**Methods:**

We conducted a systematic review of relevant English-language full-text original articles retrieved from PubMed (1961–August 2018) that had experimental or observational study design and involved adult trauma patients. Studies involving informed consent in clinical or research trials were excluded. Titles and abstracts of searched articles were reviewed and relevant data were extracted with a structured form. Results were synthesized with a narrative approach.

**Results:**

A total of 2044 articles were identified in the initial search. Only eight studies were included in the review for narrative synthesis. Six studies involved orthopedic surgeries, one involved nasal bone surgeries, and one involved trauma-related limb debridement. Only one study was conducted in an emergency department. Information recall was poor for trauma patients. Risk recall and comprehension were greater when written or video information was provided than when information was provided only verbally. Patient satisfaction was also greater when both written and verbal information were provided than when verbal information alone was provided; patients who received video information were more satisfied than patients who received written or verbal information.

**Conclusions:**

Many articles have been published on the subject of informed consent, but very few of these have focused on trauma patients. More empirical evidence is needed to support the success of informed consent for trauma patients in the emergency department, especially within the necessarily very limited time frame. To improve the informed consent process for trauma patients, developing a structured and standardized informed consent process may be necessary and achievable; its effectiveness would require evaluation. Adequately educating and training healthcare providers to deliver structured, comprehensive information to trauma patients is crucial. Institutions should give top priority to ensuring patient-centered health care and improved quality of care for trauma patients.

**Electronic supplementary material:**

The online version of this article (10.1186/s12910-019-0347-0) contains supplementary material, which is available to authorized users.

## Background

The doctrine of informed consent, which is a fundamental ethical element and legal prerequisite in contemporary medicine, has encouraged patients to become actively engaged in their own health-decision-making processes [1–4]. However, most trauma patients in the emergency department cannot choose their treating hospital or their healthcare providers. Emergency physicians have time constraints and little chance to understand patients’ preferences and values. Hence, securing patients’ informed consent is especially important to respect their autonomy and promote their well-being [2].

### Philosophy of informed consent

Two fundamental moral values are nurtured by informed consent: patient well-being and patient autonomy [1, 2]. Since ancient times, the core value of medicine has been protection and promotion of patient well-being. Physicians must devote their best efforts to promoting patients’ well-being. Healthcare providers must dedicate themselves to informing their patients about treatment risks and alternatives to help patients make treatment decisions and to promote patient well-being [2]. Autonomy literally means “self-rule” and is the principle on which the informed consent doctrine was founded [5, 6]. Autonomy may also be an instrumental value in promoting a patient’s well-being. Informed consent protects patient autonomy. Competent patients have the free will to choose or refuse treatment according to their judgments on the consequences of treatment [2].

### Fundamental elements of informed consent

Informed consent comprises several important fundamental elements, including 1) competence, 2) disclosure, and 3) voluntariness [7, 8]. A competent individual should be provided with adequate information, should be able to understand that information and weigh benefits and risks, and should make an autonomous decision without coercion [5].

A competent individual is one with decision-making capacity, which has been defined as the ability to understand information relevant to a decision and to appreciate the reasonably foreseeable consequences of a decision or lack of decision [9]. When patients in an emergency situation lack decision-making capacity, physicians may seek consent from a surrogate decision-maker such as a family member or provide treatment that would be considered appropriate and in the patient’s best interest by a reasonable person [10]. However, “where there is a choice of treatment, the treatment provided must be the least restrictive on the patient’s future choices” [11].

Disclosure refers to the process by which healthcare providers provide patients with information concerning diagnosis and treatments [12]. There are two standards for the disclosure of information in health care. The first is the “professional standard” [2, 6, 13, 14], which states that it is the duty of healthcare providers to “disclose all information that a reasonable practitioner would provide” [15]. The second standard is the “reasonable patient standard” [2, 6, 13, 16]. Based on this standard, healthcare providers must provide all information that a “reasonable” person would like to know when making a treatment decision [2]. Moreover, the Montgomery v Lanarkshire HB case provided a particular patient strand [17]. When disclosing such information, healthcare providers must discuss with their patients exactly what factors are significant for them. Healthcare providers need to enter into a process of shared decision-making in which they communicate the material risks and benefits of the available options to their patient [11, 18].

Furthermore, patients must understand the information provided by physicians to make an autonomous decision. The patient must then be allowed to make treatment decisions freely, without any coercion or duress [19]. Voluntariness refers to “a patient’s right to make treatment decisions and decisions about his or her personal information free of any undue influence” [12].

In addition to the ethical elements of informed consent, there are also legal requirements. According to legal requirements, the physician should provide explanations of procedures, possible risks and complications, benefits of procedures, and available alternatives, including the consequences of foregoing treatment [2, 15]. Although there is no universal rule concerning when and which procedures require consent and documentation, a written consent form is usually prepared for invasive procedures that have relatively higher risks in clinical practice [12, 20]. If there is no consent document for a specific procedure, physicians may generally write notes about possible risks in the chart.

### Challenges of obtaining informed consent in trauma patients

Traumatic injury is the sixth leading cause of death among all patients, and one of the leading causes of death among patients aged 25 to 44 years in Taiwan [21]. The informed consent dilemma is a profound challenge for these patients and their families. The time constraints, emotional stress, and physical pain of sudden injury impair the immediate comprehension of relevant information essential to providing consent [1, 2, 22]. Conflicts between a patient’s values and perspectives concerning treatment and those of physicians might further increase the psychological stress of patients and family members.

#### Involuntary nature of emergency care for trauma patients

Unconscious trauma victims, taken by ambulance to the emergency department, are unable to choose their treatment team. The patient may be meeting the physician for the first time, a good patient–physician relationship may not have been established, and the physician might not know the values and preferences of the patient. Therefore, for trauma patients, there is often an unavoidable coercive element, with patients unable to choose the hospital and physicians, and the priorities of both the hospital and physician may not match those of the individual. These circumstances explain the involuntary nature of emergency care in trauma patients, with patients unable to voluntarily consent to procedures. Moreover, many institutions are mainly designed and function for the general public rather than for individuals, which may limit patient autonomy and decision-making [23].

#### Consent in medical emergencies for trauma patients

There are several conditions where it is permissible not to obtain informed consent for medical treatment. First, bypassing informed consent is permissible when patients who lack the capacity (competence) to provide consent need immediate treatment to preserve life or avoid serious harm [1]. Other situations in which informed consent may not be required include “patient waiver of consent,” “public health requirements,” and “therapeutic privilege” [1, 2, 24, 25].

Informed consent need not be procured in medical emergencies “when immediate intervention is necessary to prevent death or serious harm to the patient” [26, 27]. Because of this exception, some physicians mistakenly believe that informed consent is not important when patients present to an emergency setting. However, most patients in emergency settings, including trauma patients, do not require immediate intervention to prevent death or serious harm and are competent to provide consent [2, 28]. For example, although this study does not address consent for surgical procedures, Moore et al. reported that it is likely feasible to obtain informed consent for computed tomography in over two-thirds of adult acute trauma patients [28]. Therefore, healthcare providers must attempt to obtain valid consent from trauma patients whenever feasible.

When encountering a trauma patient, emergency physicians must determine whether there is sufficient time to obtain informed consent without delaying treatment and increasing patient risks [5]. Many issues related to this decision remain controversial. For example, according to the statement of the American Medical Association, a medical emergency is a situation in which “harm from failure to treat is imminent” [26, 29, 30]. However, there is no clear definition of the level of imminent harm that constitutes a medical emergency. Physicians in these situations may have difficulty judging the necessity of informed consent.

#### Consent for incompetent patients

Patients who are severely injured, such as those in shock or who have sustained brain injuries, may not be able to participate in discussions of treatment decisions or to provide consent. When patients do not have the capacity to provide consent, physicians may make medical decisions based on the patient’s “best interest” [31], or may seek consent from patient surrogates. “Surrogate decision-makers are called upon to make decisions on behalf of incompetent patients” [10, 32]. There are special challenges for physicians to obtain valid informed consent and for surrogates to make treatment decisions on behalf of the patient’s best interest when considering emergency surgery in incompetent trauma patients. Surrogates must usually make treatment decisions within a short period of time. If patients are transferred to a remote hospital far from their families or surrogates, the process of seeking consent from surrogates may be challenging for physicians and hospitals. It remains a challenge if surrogates are unable to arrive in a timely manner to provide consent, and discussion of treatment decisions between physician and surrogate might be limited. The quality of communication may be insufficient.

Informed consent ideally is a process in which physicians build rapport and relationships with their patients and assist them in decision-making [33]. Some authors have found that patient recall about the consent process during acute illness is variable and sometimes poor, with many patients having no recollection of the process at all [34]. The issue of poor recall in trauma patients with potentially serious complications who have little time to absorb complicated information needs to be addressed to improve the consent process and increase its validity. In our clinical experience, trauma patients may have difficulty retaining information presented to them, and are therefore unable to imagine the surgery process. Therefore, a cooperative effort by healthcare providers should present critical information in an effective way, to help patients and family members gain adequate knowledge to make treatment decisions even under stressful situations.

This study aimed to explore the challenges of obtaining valid consent and methods of improving the quality of the informed consent process for surgical procedures in trauma patients. We conducted a systematic review of the informed consent process in trauma patients to answer the above questions.

## Methods

### Search strategy

A systematic review was conducted to identify relevant articles, according to the PRISMA guidelines [35]. A 27-item checklist and four-phase flow diagram were included in the PRISMA statements. The search terms applied to PubMed (1961–August 2018), included “informed consent,” “trauma/traumatic,” “polytrauma,” “injury/injuries,” “wound(s),” “laceration(s),” “fracture(s),” “rupture(s),” and “hemorrhage(s)” (Table 1). The inclusion criteria included full-text original articles with experimental or observational study design in adult trauma patients requiring consent for any surgical procedure and published with a peer-reviewed process in scholarly English-language journals. All studies had to include outcome or satisfaction evaluation. In addition, the references of the selected articles were searched by hand and reviewed. Studies concerning informed consent in clinical or research trials were excluded.

**Table 1:** Literature search strategy for PubMed

	Key words	Search results
#1	(informed consent [Title/Abstract]) AND trauma [Title/Abstract]	423
#2	(informed consent [Title/Abstract]) AND traumatic [Title/Abstract]	252
#3	(informed consent [Title/Abstract]) AND polytrauma [Title/Abstract]	7
#4	(informed consent [Title/Abstract]) AND injury [Title/Abstract]	830
#5	(informed consent [Title/Abstract]) AND injuries [Title/Abstract]	307
#6	(informed consent [Title/Abstract]) AND wound [Title/Abstract]	241
#7	(informed consent [Title/Abstract]) AND wounds [Title/Abstract]	62
#8	(informed consent [Title/Abstract]) AND laceration [Title/Abstract]	19
#9	(informed consent [Title/Abstract]) AND lacerations [Title/Abstract]	7
#10	(informed consent [Title/Abstract]) AND fracture [Title/Abstract]	227
#11	(informed consent [Title/Abstract]) AND fractures [Title/Abstract]	205
#12	(informed consent [Title/Abstract]) AND rupture [Title/Abstract]	129
#13	(informed consent [Title/Abstract]) AND ruptures [Title/Abstract]	10
#14	(informed consent [Title/Abstract]) AND hemorrhage [Title/Abstract]	248
#15	(informed consent [Title/Abstract]) AND hemorrhages [Title/Abstract]	25
#16	((((((((((((((((informed consent [Title/Abstract]) AND trauma [Title/Abstract])) OR ((informed consent [Title/Abstract]) AND traumatic [Title/Abstract])) OR ((informed consent [Title/Abstract]) AND polytrauma [Title/Abstract])) OR ((informed consent [Title/Abstract]) AND injury [Title/Abstract])) OR ((informed consent [Title/Abstract]) AND injuries [Title/Abstract])) OR ((informed consent [Title/Abstract]) AND wound [Title/Abstract])) OR ((informed consent [Title/Abstract]) AND wounds [Title/Abstract])) OR ((informed consent [Title/Abstract]) AND laceration [Title/Abstract])) OR ((informed consent [Title/Abstract]) AND lacerations [Title/Abstract])) OR ((informed consent [Title/Abstract]) AND fracture [Title/Abstract])) OR ((informed consent [Title/Abstract]) AND fractures [Title/Abstract])) OR ((informed consent [Title/Abstract]) AND rupture [Title/Abstract])) OR ((informed consent [Title/Abstract]) AND ruptures [Title/Abstract])) OR ((informed consent [Title/Abstract]) AND hemorrhage [Title/Abstract])) OR ((informed consent [Title/Abstract]) AND hemorrhages [Title/Abstract])	2044

#### Study data extraction

Two reviewers evaluated titles and abstracts of searched articles. For those studies addressing the subject of this study, the full-text version was obtained and further review was conducted. Two reviewers examined every full-text article using the selection form. If there was doubt, the two reviewers discussed the issue further and reached a consensus. If a consensus was not reached, a third reviewer was consulted.

Two reviewers used the structured extraction form (see Additional file 1: Appendix 1) to extract relevant data, including authors, country, study aim, study design, inclusion criteria, participant recruitment procedures, numbers of participants, and participant characteristics (diagnosis, gender, age, level of education, disease or injury severity, and surgery performed).

### Methodological quality assessment

The methodological quality of included articles was assessed. For nonrandomized studies, the methodological quality was assessed using the framework of the Newcastle-Ottawa Quality Assessment Scale [36, 37]. Seven domains were modified to assess the risks of bias, including case definition, representativeness of the cases, ascertainment of exposure, same method of ascertainment, nonresponse rate, selection of controls, and definition of controls. For randomized controlled trials, the methodological quality was assessed using the framework for assessing the risk of bias developed by the Cochrane Collaboration [38, 39]. Six domains were modified in the assessment, including sequence generation, allocation sequence concealment, blinding of outcome assessment, incomplete outcome data, selective outcome reporting, and other potential threats to validity. The methodological quality checklist was used (see Additional file 2: Appendix 2).

### Data synthesis

Because of the heterogeneity of methodologies, it was impossible to conduct a meta-analysis. Therefore, results were synthesized with a narrative approach.

## Results

**Fig. 1:**
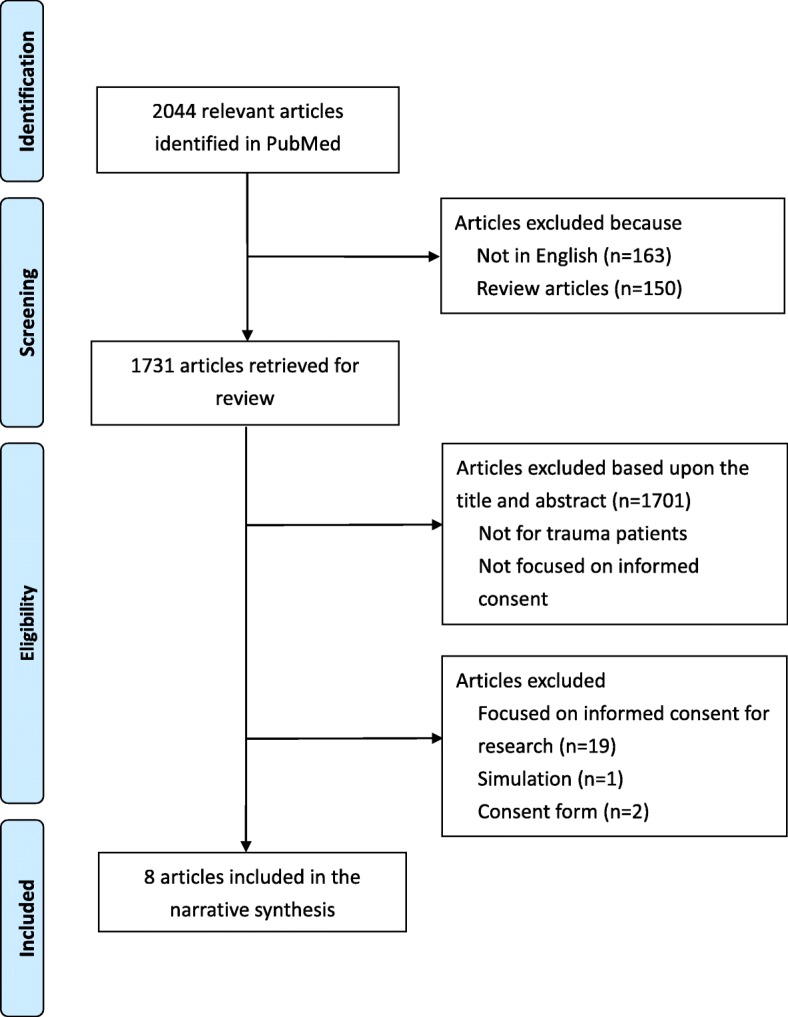
Flow chart for selection of reviewed articles

Figure 1 presents the search process in detail to identify the eligible studies for inclusion in the review. A total of 2044 articles were identified at the initial search. 163 articles not published in English and 150 review articles were excluded. 1731 articles were retrieved for review. 1701 articles not meeting the interest of the study were also excluded. Nineteen articles focusing on informed consent for research, one article conducted with simulation, and two articles auditing the consent forms, were also excluded.

The remaining eight studies on informed consent in adult trauma patients were included in the review for narrative synthesis [40–47] (Table 2). One study was conducted in the United States [40], one in Turkey [42], one in Korea [45], one in Taiwan [46], three in the United Kingdom [41, 43, 44], and one in Ireland [47]. All studies were conducted to evaluate the informed consent process in adult trauma patients. Six studies involved orthopedic surgeries [40–44, 47], one involved surgery for nasal bone fracture [45], and one involved trauma-related debridement surgery [46]. Only one study was conducted in an emergency department [46].

**Table 2:** Selected studies regarding the informed consent process for trauma patients undergoing a surgical operation

Author/year and country of publication	Study aims	Procedure/ISS^a^	Study design/number of patients/age/gender/level of education			Methods of information provided to patients	Timing/methods of evaluation/content of evaluation	Results
Rossi et al. (2004, UK)	Evaluate the effectiveness of using a videotape to give patients information a common orthopedic procedure	Procedure: Ankle fracture fixation ISS: no description	Study design: Randomized controlled trial Number of patients: Conventional group: 25 Videotape group: 23 Age, mean: Conventional group: 39.6 Videotape group: 36.1 Gender, male, n: Conventional group: 12 Videotape group: 15 Level of education, ≥High school, n: Conventional group: 19 Videotape group: 16			Conventional group: verbal Videotape group: video	Timing: Immediately after receiving information and an average of 10 weeks later Methods of evaluation: Multiple choice questionnaire Content of evaluation: Procedure Benefits Risks/complications Alternatives	Patients who received information on a videotape demonstrated a significant increase in comprehension compared to patients who received this information verbally by 40.1% on the initial evaluation and 27.2% on the follow-up evaluation. Patients with education level less and equal to the 12th grade performed 67.8% better on initial evaluation after watching the video.
Bhangu et al. (2008, US)	Compare patient recall of the consent process and desire for information between orthopedic trauma and elective patients	Procedure: Femoral neck fracture fixation, other trauma operations/elective orthopedic operations ISS: no description	Study design: Non-randomized controlled trial Number of patients: Elective group: 41 Trauma group: 40 Age, mean: Elective group: 68 Trauma group: 66 Gender: no description Level of education: no description			Elective group: verbal and leaflet Trauma group: Verbal	Timing: First post-operative day Methods of evaluation: Questionnaire Content of evaluation: Procedure Risks/complications Satisfaction with the consent process	Overall recall of complications was poor in trauma patients than in elective patients (22.4% vs 62.3%); trauma patients desire more information than elective patients (30% vs 12%). Repeated verbal explanation and additional information leaflets should be considered for trauma operation.
Sahin et al. (2010, Turkey)	Evaluate the effectiveness of the consent process and the retention of information in orthopedic patients undergoing trauma and elective surgery	Procedure: Fracture fixation/elective orthopedic operations ISS: no description	Study design: Non-randomized controlled trial Number of patients: Elective orthopedic surgery: 70 Orthopedic trauma: 72 Age, mean: 52.02 Gender, male, n: 63 Level of education, ≥High school, n: 29			Verbal and written information	Timing: Post-operative 1–3 days Methods of evaluation: Interview and questionnaire Content of evaluation: Procedure/operation Complications	Advanced age had a negative impact and level of education had a positive impact on recall of information. Trauma patients have higher rate of not recalling any potential complications, and most have not read the consent form.
Khan et al. (2012, UK)	Investigate the patient and process factors that influence consent information recall in mentally competent patients presenting with neck of femur fractures.	Procedure: Neck of femur fracture group: Femoral neck fracture fixation or hemiarthroplasty Total hip replacement group: Total hip replacement ISS: no description	Study design: Non-randomized controlled trial Number of patients: Neck of femur fracture group: 50 Total hip replacement group: 50 Age, mean: Neck of femur fracture group: 79.3 Total hip replacement group: 66.6 Gender, male, n: Neck of femur fracture group: 11 Total hip replacement group: 24 Level of education: no description			Verbal and consent form	Timing: First post-operative day Methods of evaluation: formalized questionnaire proforma for semi-structured interview Content of evaluation: Procedure Risks	Trauma patients had poor recall of information provided at the time of consent. Twenty-six percent of trauma patients recalled correctly the surgery and 48% recalled the risks and benefits. Pre-printed consent form, information sheet, and visual aids may improve retention and recall.
Smith et al. (2012, UK)	Assess whether written information improves trauma patient’s recall of the risks of surgery	Procedure: Upper and lower limb fracture fixation ISS: no description	Study design: Randomized controlled trial Number of patients: Verbal group: 50 Verbal and written group: 50 Age, mean: Verbal group: 57.0 Verbal and written group: 67.2 Gender: male, n Verbal group: 23 Verbal and written group: 20 Level of education: no description			Verbal group: verbal Verbal and written group: Verbal and written	Timing: 1–17 days (mean 3.2 days) Methods of evaluation: Questionnaire Content of evaluation: Risks Satisfaction with the consent process	Risk recall and satisfaction improved when patients receiving written and verbal information compared to verbal information alone. Overall recall of risks was poor in patients receiving oral information only than in patients receiving written and verbal information (mean questionnaire score: 41% vs 64%). Ninety percent of patients preferred to having written and verbal information.
Kim et al. (2018, Korea)	Investigate whether delivery of information through a mobile application is more effective than through only verbal means and a paper	Procedure: Close reduction for nasal bone fracture ISS: no description	Study design: Randomized controlled trial Number of patients: Experimental group: 29 Control group: 28 Age, n (%):			Experimental group: a mobile application with verbal descriptions and paper permission Control group: verbal descriptions and paper permission	Timing: follow-up visit 4 weeks later Methods of evaluation: Open question Content of evaluation: Surgical risks	The mean number of recall surgical risks was 1.72 ± 0.52 in the experimental group and 1.49 ± 0.57 in the control group. Age, gender, and level of education had no influence on the number of recall surgical risks. A mobile application could contribute to the efficient delivery of information during the informed consent process.
				Experimental	Control			
			19–29	11 (37.9)	10 (35.7)			
			30–39	7 (24.1)	8 (28.6)			
			40–49	6 (20.7)	8 (28.6)			
			50–59	4 (13.7)	2 (7.1)			
			> 60	1 (3.4)	0			
			Gender: male, n Experimental group: 22 Control group: 20 Level of education, ≥High school, n: Experimental group: 28 Control group: 26					
Lin et al. (2018, Taiwan)	Investigate whether, in the emergency department, educational video-assisted informed consent is superior to the conventional consent process, to inform trauma patients undergoing surgery	Procedure: trauma-related debridement surgery ISS, ISS > 4, %: Control group: 23.6 Intervention group: 18.6	Study design: Randomized controlled trial Number of patients: Control group: 72 Intervention group: 70 Age, %:			Control group: written Intervention group: video	Timing: Before and immediately after receiving information Methods of evaluation: Multiple choice questionnaire Content of evaluation: Procedure Benefits Risks/complications Alternatives Satisfaction with the consent process	Mean knowledge scores were higher in the intervention than in the control group (72.57 ± 16.21 (SD)) vs (61.67 ± 18.39). The intervention group had significantly greater difference in knowledges (coefficient: 7.646, 95% CI: 3.381–11.911). Age, injury severity score, and baseline knowledge score had significantly influence on the differences in knowledge scores. Both the educational approach and severity of injury may have an impact on patient understanding during the informed consent process in an emergency environment. Video-assisted informed consent may improve the understanding of surgery and satisfaction with the informed consent process for trauma patients.
				Control	Intrvention			
			< 20	2.8	12.9			
			20–29	31.9	35.7			
			30–39	23.6	12.9			
			40–49	16.7	17.1			
			50–59	18.1	14.3			
			60–69	2.8	4.3			
			> 69	4.2	2.9			
			Gender, male, %: Control group: 59.7% Intervention group: 51.4% Level of education, ≥High school, %: Control group: 81.9% Intervention group: 88.6%					
Clarke et al. (2018, Ireland)	Investigate the effect of a standardized consenting process with patient take-home information sheets on patient information recall	Procedure: Wrist manipulation and K-wiring ISS: no description	Study design: Non-randomized controlled trial Number of patients: No take-home information group: 50 Take-home information group: 47 Age, mean: 44 years (range, 17–81 years) Gender, male, n: 52 Level of education: no description			Written/written and take-home information sheet	Timing: Immediately and 1 day later Methods of evaluation: Questionnaire Content of evaluation: Procedure Anesthesia Risks Alternatives	Baseline scores were low on initial questioning. Information retention after 24-h was significantly decrease (mean, 8.94 versus 7.98), but rose when standardized forms were provided. Significant lower scores were noted among those participants without receiving written information (mean, 9.542 versus 6.449).

^a^ISS: injury severity score

Four studies were conducted with an observational study design [41–43, 47]; the other four studies were conducted with an experimental study design [40, 44–46]. Only one study described injury severity scores [46]. The number of included patients was 48, 81, 142, 100, 100, 57, 142, and 97 respectively. Four studies provided information on patient age, gender, and level of education [40, 42, 45, 46]. Three studies provided information on patient age and gender alone [43, 44, 47]. One study provided information on patient age only [41]. In five studies, verbal and written/leaflet information were provided to patients [41–44, 47]; in one study, verbal and video information were provided to patients [40]; in one study, verbal description and paper permission with or without a mobile application were provided [45]; and in one study, written or laptop-based video information was provided to patients [46]. The timing of patient evaluation was immediately after receiving information and an average of 10 weeks later [40], on the first postoperative day [41, 43], on postoperative days 1 to 3 [42], on postoperative days 1 to 17 (mean 3.2 days) [44], at a follow-up visit 4 weeks after information was provided [45], before and immediately after receiving information [46], and immediately and one day later [47]. Five studies used a questionnaire [40, 41, 44, 46, 47], two used interview and questionnaire [42, 43], and one used open questioning to evaluate recall of surgical risks [45]. Two studies developed a multiple-choice questionnaire to evaluate the understanding of trauma patients about their surgery [40, 46]; the other six asked patients to recall the name of the procedure and risks or complications of the surgery [41–45, 47]. Two studies evaluated patient satisfaction with the consent process [41, 46].

The results revealed that recall of diagnosis, surgical procedures, risks and benefits, and complications was poorer for trauma patients than for patients undergoing elective surgery [41–43]. Risk recall and comprehension were greater when written or video information was provided than when information was provided only verbally [40, 44]. Trauma patients had better comprehension with video education than with written information [46]. Satisfaction was greater when patients received both written and verbal information than when patients received verbal information alone [44]. Satisfaction was also greater when patients received video information than when patients received written information [46]. Trauma patient age, level of education, injury severity, and baseline knowledge may influence comprehension during the informed consent process [40, 42, 46]; however, one study reported that these factors did not influence recall of information [45]. A portable computer or mobile application may be helpful in the delivery of information and may improve patients’ comprehension [45, 46].

### Assessment of methodological quality

The assessment of methodological quality is presented in Table 3. Three of the nonrandomized studies [41, 42, 47] adequately described the case definition and exposure using the same method for both groups and reported the nonresponse rate; however, three studies had a risk of bias because the selected participants may not have represented the overall population. In one nonrandomized study [43], which was conducted with case-control study design, the case definition was adequately described, the selected participants adequately represented the population, the selection and definition of controls were stated, and the nonresponse rate was reported. Among the randomized controlled studies [40, 44–46], four adequately described the incomplete outcome data, selective outcome reporting, and other potential threats to validity. Three studies did not describe the sequence generation or allocation sequence concealment [40, 44, 45], and two did not report the allocation sequence concealment or blinding of outcome assessment [44, 45].

**Table 3:** Methodological quality assessment

	Bhangu et al. (2008, US)	Sahin et al. (2010, Turkey)	Khan et al. (2012, UK)	Clarke et al. (2018, Ireland)	Rossi et al. (2004, UK)	Smith et al. (2012, UK)	Kim et al. (2018, Korea)	Lin et al. (2018, Taiwan)
Non-randomized studies								
Case definition	✓	✓	✓	✓				
Representativeness of the cases	X	X	✓	X				
Ascertainment of exposure	✓	✓		✓				
Same method of ascertainment	✓	✓		✓				
Non-response rate	✓	✓	✓	✓				
Selection of controls			✓					
Definition of controls			✓					
Randomized controlled trials								
Sequence generation					X	✓	✓	✓
Allocation sequence concealment					X	X	X	✓
Blinding of outcome assessment					✓	X	X	✓
Incomplete outcome data					✓	✓	✓	✓
Selective outcome reporting					✓	✓	✓	✓
Other potential threats to validity					✓	✓	✓	✓

## Discussion

### Main findings

A systematic review was conducted to evaluate the informed consent processes for surgical procedures in trauma patients. Many articles have been published on the subject of informed consent; however, very few of these have focused on informed consent in trauma patients. Although 150 review articles were identified in our research, none addressed this issue.

The investigators identified eight studies for analysis, and found that trauma patients had poor recall of diagnosis, surgical procedures, risks and benefits, and complications. Written information, pamphlets, or videos had positive effects on patients’ understanding and satisfaction. Written information may improve patients’ knowledge more than oral information, and video information may improve patients’ comprehension more than written information. The investigators posit that video or interactive media improve patients’ comprehension and satisfaction. Furthermore, we found that many factors may affect patients’ comprehension during the informed consent process, including age, level of education, injury severity, and baseline knowledge. The methods of evaluating patients’ knowledge and comprehension varied, and the timing of this evaluation also was very different across studies. To our knowledge, ours is the first systematic review to study informed consent in trauma patients.

Treating trauma patients, especially severely injured patients, is beyond the scope of traditional emergency departments. Management of trauma patients necessitates dedicated team work and appropriate communication with patients or their proxies. A multidisciplinary trauma team that includes trauma surgeons, emergency medicine physicians, anesthesiologists, neurosurgeons, and orthopedic surgeons is essential. Comprehensive emergency medical services must be readily available for patients with serious trauma. Our research has shown that trauma patients have poor recall of information during the informed consent process. We attribute their poor recall to several factors. First, physical pain and emotional stress may have impacts on the informed consent process. Some studies reported that patients undergoing emergency surgery do not fully read or understand the consent form [48]. These patients reported that they felt they had no choice about signing the consent form, regardless of its content, and felt fearful when asked to sign it. However, patients who had read and agreed with the consent form and whose healthcare providers had ensured that they understood it felt more satisfied than those who had not experienced this [48]. Emergency surgery is frequently required by trauma patients; however, not all such patients require emergency surgery. Moreover, although many patients requiring emergency surgery or being managed in emergency settings share similar characteristics and face similar scenarios, we believe that the informed consent process is more problematic for trauma patients than for patients in other categories. Each trauma patient has unique characteristics and faces a unique scenario. They may have diverse types of injuries and those injuries may be complex and vary widely in severity, especially in patients who have sustained severe or multiple trauma. The content of information that should be imparted for different conditions may differ considerably and delivery of complete information may be challenging. All these factors may influence trauma patients’ comprehension and the informed consent process. Five studies that we reviewed were on orthopedic patients, one on individuals with nasal bone fractures, and another on individuals requiring trauma-related limb surgery. All these studies were conducted on less complicated, relatively stable trauma patient cohorts. Only one study was conducted in an emergency department. This may reflect the challenges of obtaining valid informed consent and conducting relevant research in trauma patients. Therefore, healthcare providers should more strongly prioritize patients’ comprehension. Obtaining valid informed consent from trauma patients should be ensured and this may well require a dedicated informed consent process.

Although informed consent is a critical issue for physicians, not all physicians recognize its importance in their clinical duties [15]. In some studies, the administration and documentation of informed consent for surgical care were inadequate [49, 50]. Poor documentation of risks and complications revealed that patients might not have received appropriate information and that the consent might not have been valid [51, 52]. Another study revealed that the provision of pre-operative counseling for surgical informed consent in obstetric and gynecologic surgeries might not be comprehensive and standardized [53].

In our research, two studies reported that informed consent for surgical procedures was obtained by residents or chief residents [40, 46]. Residents may not have enough clinical experience to anticipate unforeseen treatment complications and risks. Furthermore, some residents may not have adequate communication skills to explain information in detail [54–56]. The information provided to patients may not be complete. Hence, patients’ needs may not be properly met by current principles of consent to treatment, particularly in emergency circumstances. One study recommended that a specific training program on obtaining consent for common orthopedic trauma procedures should be developed for junior doctors [57]. Moreover, if a patient refuses a life-saving procedure in an emergency situation, junior residents may lack the confidence to handle the ethical dilemma [58].

One of the included studies reported that although the consent forms obtained from patients were adequate, trauma patients had poor information-recall scores. That study recommended preprinted consent forms, information sheets, and visual aids to improve patients’ retention and recall [43]. One study also revealed that preprinted consent forms containing risks and benefits might improve the standard of informed consent [59]. Another study revealed that the use of a procedure-specific label could improve the informed consent process and documentation as well as the communication between medical staff and patients [60]. Although many hospitals have informed consent forms that include explanations of procedures, risks, and alternatives in detail, it should not be presumed that all patients can understand all the information provided on their case. Notably, one study reported concerns about the quality of informed consent forms, and found that the consent forms in use had communication deficiencies, particularly in describing risks [61]. Moreover, such written consent is generally designed to protect clinicians and hospitals from litigation rather than for the benefit of patients [15, 62]. This fact is not concordant with the core values and principles of informed consent, and may be harmful to the patient–physician relationship. Therefore, physicians and institutions should develop strategies to improve the informed consent process in the best interests of patients.

### Strategies for improving the consent process in emergency settings

#### Shared decision-making

The process of obtaining consent has been described as the most fundamental element in building a successful physician–patient relationship [12]. As Bernat and Peterson have reported, “all surgeons should conceptualize consent not as a discrete event but as an ongoing bidirectional process of communication, education, question-answering, and listening with the patient or surrogate that proceeds through the continuum of care” [63]. In shared decision-making, the physician serves as the patient’s partner. Physicians provide patients with their professional knowledge about diagnosis, treatment options, prognosis, and possible risks and benefits, and frequently propose treatment recommendations. Patients may provide physicians with information about their own values, life goals, and treatment preferences to help physicians recommend a proper approach [63–65].

Informed consent should be regarded as a continuing conversation and discussion between patient and physician throughout the patient’s care [13, 25, 63, 66, 67]. Patients may change their minds about treatment decisions at any time in response to changes in their condition and to additional information they may receive. Thus, “informed consent is also viewed as a process of patient-centered decision-making” [63].

Schwarze et al. proposed a best-case/worst-case framework for physicians communicating with patients and families during medical decision-making [68]. Physicians may provide an overall picture for patients and families about all potential choices, what the best-case and the worst-case scenarios may be, and where the patient may lie on the continuum. The framework provides a feasible tool for physicians to align patients’ comorbidities, values, and preferences, and to help patients make treatment decisions. We believe that this framework may also be applied to trauma patients.

#### Improving patient comprehension

Many strategies have been adopted to achieve better patient understanding, including use of illustrative materials, leaflets and pamphlets, video descriptions, interactive computer programs [69–78], and “repeat back to me” or testing with feedback strategies [79–81]. Such strategies have both advantages and limitations. In our research, written information was reported to be helpful for trauma patients [41, 44]. However, such material usually requires active collaboration and compliance on the part of the patient, and transfer of knowledge concerning procedures and risks to the patient is often limited. Some studies indicate that a significant number of patients do not even read the consent form before signing [82], while one study concluded that trauma patients often need repeated verbal explanations of procedures and potential complications rather than written information alone [41].

Using video or multimedia modalities to educate patients and assist informed consent seems to produce satisfactory results. Several studies have shown that using a video-assisted method to educate patients resulted in better patient satisfaction and improved patient knowledge of procedures and risks [74, 77, 83, 84]. Some studies also found that the use of educational videos can reduce physician counseling time [75, 85]. Two of the included studies had introduced the use of videos for trauma patient education, with promising results [40, 46].

Because most of these studies focused on elective procedures or surgeries, and because the problem of patient understanding and information retention may be greater with trauma procedures and surgeries, institutions should develop effective educational tools to foster the informed consent process. Delivering such information is fundamental, as is the provision of supportive materials [86]. Therefore, it is crucial to standardize the communication process for patients and their families to improve the effectiveness and efficiency of the communication process. Using the information aids mentioned above could reduce the burden of communication between physicians and patients, and could improve the consent process by delivering standardized information [46].

“Most patients have a positive attitude toward receiving information” [87]. However, at what level necessary information becomes “sufficient” is an important determinant of patient satisfaction; more attention should be focused on this area [88]. Nnabugwu et al. reported that efforts should be made to ensure that consent information, including the nature of the disease condition, the nature of planned procedures, and risks, are satisfying from the patient’s viewpoint [89]. Some studies recommended that it was crucial to use the scientific method to define the core information for informed consent [90, 91] and to involve patients in the development process [91].

One included study used a mobile smartphone [45] and one used a laptop computer to deliver information [46]. The weight and size of modern electronic tools have previously limited their application in emergency settings. However, recent advances in portable and tablet computer technology provide good opportunities for improving patient education for surgery [22]. Innovative, less bulky portable computers have larger screen displays, larger memory storage, and good image resolution, and can more easily deliver educational information and high-quality videos. The use of such innovative computer technology may help with preoperative education in trauma patients requiring emergency surgery. Such technological tools, however, should never replace interaction between the physician and the patient, and patients should be given an opportunity to ask questions and voice their concerns.

Obtaining adequate informed consent in the emergency department is a challenging and time-consuming process. Because of the involuntary nature of emergency care, informed consent is the only way to respect patients’ autonomy. Providers must communicate complicated medical information to patients to help them make informed decisions. In most emergency settings, the time constraints and stress as well as patient distress caused by pain or other acute symptoms result in patients and their families having difficulty understanding the significant information needed to provide valid informed consent. Hence, the use of video to assist the informed consent process for surgery may offer a practical solution. The use of video to support preoperative education may improve both patient satisfaction and comprehension.

The importance of effective and efficient preoperative education and communication as well as the entire consent process before emergency surgery should not be underestimated. A good consent process will dramatically increase the satisfaction of trauma patients undergoing emergency surgery. To obtain informed consent effectively and efficiently, a comprehensive tool and a standardized consent process should be developed in emergency settings for trauma patients and their families.

### Implications for future research

Informed consent is very important in trauma patients but has rarely been studied in this population. Further studies are needed on the details of the informed consent process in trauma patients, including the determinants affecting the process and the satisfaction of trauma patients. Further research is needed to confirm the effectiveness of different information-delivery methods in trauma patients, to facilitate development of the most effective strategy for the process.

Furthermore, further exploration is needed on providing adequate education and training to healthcare providers so that they can deliver structured and comprehensive information to trauma patients in a timely manner, establishing a good patient–physician relationship and building trust.

Moreover, informed consent might be waived for patients who are in medical emergencies. Further research is needed to explore how many unconscious trauma patients undergo emergency surgery without informed consent or surrogate consent, and how healthcare providers define such medical emergencies. More research is needed about the relationship between patient outcomes and their decision-making process.

### Implications for policy and practice

This review revealed that research on informed consent for trauma patients, including the best tools to convey complete information on possible risks and treatments, is rare. This lack of research might greatly limit patients’ ability to obtain sufficient information concerning risks and benefits, information that would enable them to make autonomous decisions that respect their values and really benefit them. We recommend that appropriate information aids should be provided so that healthcare providers do not provide only verbal information with imprecise terms to describe risks and outcomes (such as low, uncommon, etc.). Patients provided with such imprecise information might overestimate or underestimate the possible harm.

Computerized and interactive programs can provide patients with tailor-made, individualized information to help them comprehend all necessary information in a very short time frame. We believe that information aids have many advantages for trauma patients. The model of shared decision-making is currently favored, especially when there are two or more options for treating one condition, each with different risks and benefits, with no single best treatment, and in which professional consensus is not yet achieved. For instance, the options for treating splenic laceration include surgical treatment (splenectomy or splenorrhaphy) and nonsurgical treatment (conservative or transarterial embolization). Each option has its own risks and benefits. In some circumstances, healthcare providers must discuss these options with patients before making treatment decisions.

Our study has several strengths. The search strategy was comprehensive. As far as we know, no other review study has focused on this topic. Our review also has several limitations. The searched articles are rare, and meta-analysis and quantitative analysis were not possible because of the heterogeneity of the data. Because the articles are rare and thus the study sample is relatively small, publication bias is possible. The results reveal a positive effect, but negative effects are possible in unpublished studies.

## Conclusions

Many articles have been published on the subject of informed consent, but few have focused on the population of trauma patients. More empirical evidence is needed to support the success of informed consent for trauma patients in the emergency department, especially for patients who must provide consent within a very limited time frame. To improve the informed consent process for trauma patients, developing a structured and standardized informed consent process may be necessary and achievable; its effectiveness would require evaluation. Providing adequate education and training to healthcare providers in delivering structured and comprehensive information to trauma patients is crucial. Institutions should give top priority to ensuring patient-centered health care and improved quality of care for trauma patients.

## Additional files

Additional file 1: **Appendix 1.** Data extraction form. (DOCX 15 kb)

Additional file 2: **Appendix 2.** Methodological quality checklist. (DOCX 13 kb)
