# Supplementary material for: How to effectively obtain informed consent in trauma patients: a systematic review
Source: BMC Med Ethics. 2019 Jan 23;20:8. doi: 10.1186/s12910-019-0347-0 (PMC6343333; doi:10.1186/s12910-019-0347-0)
Supplement: Supplementary file 1 — Appendix 1. Data extraction form. (DOCX 15 kb) [file 12910_2019_347_MOESM1_ESM.docx]

Appendix 1 Data extraction form

| **Author(s)** |  | | | | | | |
| --- | --- | --- | --- | --- | --- | --- | --- |
| **Published year** |  | | | | | | |
| **Country of publication** |  | | | | | | |
| **Funding** |  | | | | | | |
| **Study aim** |  | | | | | | |
| **Study design** | □RCT | | | | | | |
|  | □Cross-sectional study | | | | | | |
|  | □Others | | | | | | |
| **Inclusion criteria** |  | | | | | | |
| **Exclusion criteria** |  | | | | | | |
| **Number of participants** | Participants screened: | | | | | | |
|  | Participants enrolled: | | | | | | |
|  | Participants in the intervention group: | | | | | | |
|  | Participants in the control group: | | | | | | |
|  | Participants loss of follow-up | | | | | | |
| **Participant characteristics** | Age: | | mean | | | median | |
|  | Gender: | | male (n/%) | | | female(n/%) | |
|  | Ethnicity: | |  | | | | |
|  | Socio-economic status: | |  | | | | |
|  | Acute condition | |  | | | | |
|  | Procedures or operations | |  | | | | |
| **Study setting/department** |  | | | | | | |
| **Methods of information provided** | Intervention | □verbal | | □written | □video/multimedia | | □others |
|  | Control | □verbal | | □written | □video/multimedia | | □others |
| **Timing of evaluation** |  | | | | | | |
| **Methods of evaluation** | □Questionnaire | | | | | | |
|  | □Interview | | | | | | |
|  | □Others | | | | | | |
| **Outcome measurement** | □Knowledge/comprehension | | | | | | |
|  | □Satisfaction | | | | | | |
|  | □Others | | | | | | |
| **Results** | RCT | | | | | | |
|  | Intervention | | | | | | |
|  | Control | | | | | | |
|  | Details | | | | | | |
|  | Cross sectional | | | | | | |
|  | Details | | | | | | |
|  | Others | | | | | | |
|  | Details | | | | | | |
