# Supplementary material for: How to effectively obtain informed consent in trauma patients: a systematic review
Source: BMC Med Ethics. 2019 Jan 23;20:8. doi: 10.1186/s12910-019-0347-0 (PMC6343333; doi:10.1186/s12910-019-0347-0)
Supplement: Supplementary file 2 — Appendix 2. Methodological quality checklist. (DOCX 13 kb) [file 12910_2019_347_MOESM2_ESM.docx]

Appendix 2 Methodological quality checklist

| **Non-randomized studies** | | | |
| --- | --- | --- | --- |
| Was the case definition adequate? | □Yes | □No | □Unclear |
| Was the representativeness of the cases adequate? | □Yes | □No | □Unclear |
| Was the ascertainment of exposure adequate? | □Yes | □No | □Unclear |
| Was the same method of ascertainment used? | □Yes | □No | □Unclear |
| Was the non-response rate reported? | □Yes | □No | □Unclear |
| Was the selection of controls adequate? | □Yes | □No | □Unclear |
| Was the definition of controls adequate? | □Yes | □No | □Unclear |
| **Randomized controlled trials** | | | |
| Was the allocation sequence adequately generated? | □Yes | □No | □Unclear |
| Was the allocation adequately concealed? | □Yes | □No | □Unclear |
| Was there any blinding of outcome assessment? | □Yes | □No | □Unclear |
| Was incomplete outcome data adequately described? | □Yes | □No | □Unclear |
| Had the study selective outcome reporting? | □Yes | □No | □Unclear |
| Were there other threats to validity | □Yes | □No | □Unclear |
